# Opportunities and challenges of using a health information system in adolescent health management: A qualitative study of healthcare providers’ perspectives in the West Bank, occupied Palestinian territory

**DOI:** 10.1371/journal.pone.0307207

**Published:** 2024-08-22

**Authors:** Aisha Shalash, Niveen Abu-Rmeileh, Dervla Kelly, Khalifa Elmusharaf

**Affiliations:** 1 Institute Community and Public Health, Birzeit University, Birzeit, West Bank, Palestine; 2 School of Medicine, University of Limerick, Limerick, Ireland; 3 Public Health Program, University of Birmingham, Dubai, United Arab Emirates; Zarqa University, JORDAN

## Abstract

**Background:**

Adolescents are a critical demographic facing unique health challenges who are further impacted in humanitarian settings. This article focuses on the urgent need for a structured health information system (HIS) to address the gaps in data availability and evidence-based interventions for adolescent health. The study aims to identify opportunities and challenges in utilizing the HIS to enhance adolescent health in the West Bank by gathering insights from healthcare providers.

**Methods:**

Semi-structured key informant interviews were conducted with participants involved in the HIS regarding adolescent health in the West Bank. They were selected by purposive sampling. Nineteen interviews were conducted between July and October 2022, and thematic analysis was carried out using MAXQDA software.

**Results:**

The opportunities identified were the small-scale victories the participants described in building the HIS for adolescent health. These included institutional and individual capacity building, digitalizing parts of the HIS, connection fragmentation of adolescent health activities, multi-sectoral collaboration, reorienting services based on health information, working with limited resources, enhancing community engagement to encourage ownership and active participation, and taking strategic actions for adolescents for information. The challenges were the high workload of staff, lack of health information specialists, limited resources, lack of a unified system in data collection, lack of data on essential indicators, data quality, data sharing, and data sources and use.

**Conclusion:**

This study showed the potential of the HIS with capacity building, digitization, and collaborative initiatives; it also suffers from issues like staff shortages, non-standardized data collection, and insufficient data for essential indicators. To maximize the impact of the HIS, urgent attention to staff shortages through comprehensive training programs, standardization of data collection systems, and development of a unified core indicator list for adolescent health is recommended. Embracing these measures will allow the HIS to provide evidence-based adolescent health programs, even in resource-constrained and complex humanitarian settings.

## Introduction

Adolescents, defined by the World Health Organization (WHO) as individuals aged 10 to 19, represent the link between childhood and adulthood, experiencing unique health and well-being challenges dissimilar from those of adults [1]. This transitional period includes the onset of puberty and other significant developmental milestones, such as experiencing menarche [2]. Approximately 90% of the global adolescent population resides in low- and middle-income countries, where they often bear a disproportionate burden of health-related issues compared to their counterparts elsewhere [3, 4]. Some of the challenges they face are risky behaviors, violence, substance abuse, and cyberbullying [5].

Now, imagine the compounding impact of these challenges and other challenges that occur in humanitarian settings. Humanitarian settings are man-made conflicts, civil wars, or natural disasters where the affected populations cannot cope without external assistance [6]. In humanitarian settings, you see some of the same risk factors, such as substance abuse and violence, but are also burdened with factors such as infectious diseases, mental health issues, lack of access to education, displacement, crowding, and limited preventative healthcare [7]. This is currently being seen in the Gaza strip, as the war has allowed for limited preventative health care, lack of access to education, displacement, and crowding for the majority of the population [8]. What compounds the issue is the scarcity of comprehensive data and indicators to track their health status and needs [6]. The unpredictable nature of adolescent needs, resource limitations, increased internal and external migration, and the proliferation of humanitarian organizations in these settings further exacerbate the situation [9].

As more organizations intervene, implementing various programs and policies while collecting data, a structured and unified health information system (HIS) becomes vital. An HIS is an integral part of a comprehensive health system focused on collecting, processing, and reporting data to inform policy-making, programs, interventions, and research [10]. Such a system is essential for ensuring data quality and utilization [11]. Reliably collected data has been vital in revealing critical insights, such as the fact that most adolescent deaths are due to road traffic injuries [12]. However, it’s critical to acknowledge that previous interventions in adolescent health have often been of limited duration and quality, particularly in the domains of mental health, immunizations, and substance abuse. Moreover, many of these interventions have been predominantly conducted in high-income countries, making their applicability to marginalized settings questionable [13]. Transitioning from the broader context of global HISs to the specific challenges faced in the West Bank, particularly within the realm of adolescent health, emphasizes the pressing need for targeted interventions and a comprehensive understanding of the healthcare landscape in this region. While adolescents globally face significant health challenges, those in the West Bank encounter unique difficulties due to prolonged conflict and political instability [14]. These conditions make common adolescent health issues harder to treat and complicate effective healthcare delivery [15].

Roughly 20% of the population of the West Bank are adolescents [16]. Despite some efforts to address these challenges, a comprehensive understanding of adolescent health in the West Bank, specifically in the context of a chronic humanitarian crisis, remains elusive [17]. There are available interventions for mental health, sexual and reproductive health, and nutrition, but data availability is not clear to show these interventions are what is needed. This shows the urgent need for a structured HIS to address the gaps in data availability and evidence-based interventions for adolescent health in similar settings. Against the backdrop of this chronic humanitarian crisis, our study seeks to identify healthcare providers’ perspectives on the opportunities and challenges in leveraging the HIS to enhance adolescent health and services in the West Bank. Healthcare providers witness firsthand the complexities and barriers in delivering care [18]. Therefore, their insights are critical for developing an HIS that is comprehensive and responsive to adolescent health needs in the West Bank. Their perspective will allow us to determine the successes and barriers of the HIS based on their experiences. They can effectively inform of the challenges and barriers to delivering health services to adolescents. This includes issues related to data collection, coordination among different healthcare providers, and integrating HIS into routine practices. Their experiences and insights are invaluable for informing evidence-based policies and strategies tailored to the region’s needs. It will allow us to look beyond theoretical HIS improvements and recommend practical solutions for implementation. To our knowledge, this is the first study to consider adolescent health when looking at the HIS in the West Bank. This study aims to examine healthcare providers’ perspectives on the HIS in the West Bank and identify critical gaps in our current understanding of the adolescent HIS.

## Methods

The research employed a qualitative content analysis approach, conducting semi-structured key informant interviews with participants involved in the HIS regarding adolescent health and services in the West Bank.

### Context the West Bank

This study unfolds in the complex context of the West Bank, a region within the occupied Palestinian territory. Here, the Palestinian healthcare system is composed of four significant providers: the Palestinian Ministry of Health (PMoH), the United Nations Relief and Works Agency for Palestine Refugees (UNRWA), national and international non-governmental organizations (NGOs), and the private sector [19]. The PMoH plays a central role as the primary healthcare provider and data aggregator, employing the Health Management Information System Avicenna in health facilities and District Health Information Software 2 (DHIS2) in primary healthcare clinics across the country [20, 21]. Each provider has their own HIS and does not communicate with each other, leaving the HIS fragmented. Informed decision-making within the PMoH heavily relies on data collected from population-based surveys like the Multiple Indicator Cluster Survey (MICS) and surveys conducted by various NGOs operating in the region [22].

### Mapping, sampling, and selecting key participants

Semi-structured key informant interviews were conducted with participants involved in the HIS regarding adolescent health and services in the West Bank. The researchers conducted a previous mapping of all organizations involved in adolescent health. Using this list, participants were selected by purposive sampling. They were directly approached if recognized for working in an organization known to serve adolescent health or known to be involved in the HIS of their organizations. Participants were chosen based on their expertise in HISs or experience working with adolescents. They were contacted by phone, and subsequent correspondence was sent by email. Interviews were done in the workplace of participants, and only researchers and participants were present at the time of the interviews.

### Data collection

Semi-structured interview questions were adapted from three popular HIS assessment tools. These tools are the Performance of Routine Information System Management (PRISM) tools by MEASURE Evaluation [23], the Center for Disease Control (CDC) surveillance system guidelines [24], and the Health Metrics Network assessment tool [25]. Using these tools, all researchers built a topic-based interview guide: AS, NA, DK, and KE. It was built in English, translated into Arabic, and then back into English by two researchers, NA and KA, separately, fluent in both languages to ensure validity. The interview guide can be found in the S1 File. Interviews were recorded and conducted in Arabic or English, depending on the participant’s preference. The transcription was done in Arabic. AS conducted interviews; she is a current PhD candidate with a master’s degree in public health. Interviews were conducted until the researchers AS and KE felt information saturation was reached and no new information was generated from the last interviews.

#### Data analysis

Thematic analysis [26] was used to analyze the interviews, and analysis was done in English. The analysis process was guided by an interview guide structured around key themes identified a priori, including resources, indicators, data quality, data dissemination and use, and information products. This guide served as a foundational framework for exploring the perspectives of healthcare providers on the HIS for adolescent health in the West Bank. Codes were systematically generated based on predetermined themes. Similar codes were grouped to form sub-themes. For instance, under the overarching theme of "resources," sub-themes emerged, such as "financial constraints" and "staffing challenges." The coding process was iterative, and it was done after each interview. This allowed for constantly refining the codebook as new insights and perspectives emerged. Questions in subsequent interviews were adjusted based on the evolving analysis to accommodate new insights and perspectives that emerged throughout the data analysis process. Two researchers, AS and KE, used MAXQDA software for the thematic analysis [27]. This software facilitated the organization, retrieval, and visualization of coded data, enhancing the analysis’s efficiency and transparency. MAXQDA enabled a systematic exploration of patterns and relationships within the data, contributing to the transparency of our analytical process. Regular team discussions were conducted to maintain reflexivity, and researchers’ thoughts and preconceptions were documented through journaling. Both researchers, AS and KE, brought distinct yet complementary backgrounds to the analysis. With prior experience in HISs in the West Bank, AS provided contextual insights, while KE’s expertise in qualitative research and HISs contributed to methodological rigor. Any discrepancies or disagreements in coding and theme identification were addressed through discussion and consensus among the research team. Additionally, COREQ, the consolidated criteria for reporting qualitative studies, guided reporting findings, can be found as S2 File [28].

### Ethical considerations

This study was approved by the Ethical Research Committee of the Institute of Community and Public Health at Birzeit University, reference number 2022 ((4–1)). Before participating, every participant provided oral consent, understanding that they had the right to discontinue the interview at any point. Interview recordings commenced only after obtaining participant consent, and participants could halt the recording at any time if necessary. Furthermore, participants were assured that their interviews would remain confidential to the greatest extent possible, safeguarding their anonymity.

## Results

Nineteen interviews were conducted between July 2022 and October 2022, and the length of the interviews ranged from 22 to 94 minutes. There were seven males and 12 females; nine were government employees, five worked for local NGOs, and five were from international NGOs. Health providers, directors, statisticians, data collectors, and project managers were some of the interviewees’ positions. Due to anonymity, we could not identify exactly which organization and position the participant is affiliated with, other than the categorization of governmental, local non-governmental, and international non-governmental organizations and years of experience. This can be found in Table 1.

**Table 1:** Characteristics of participants: Interview length and organization type.

Identification (ID) Number	Interview Length (min)	Organization Type	Years of Experience
1	81	Governmental	28 years
2	42	Local NGO	12 years
3	27	Local NGO	14 years
4	35	Local NGO	33 years
5	39	International NGO	20 years
6	58	Local NGO	14 years
7	47	Governmental	15 years
8	22	Governmental	22 years
9	30	Governmental	17 years
10	48	Governmental	14 years
11	38	Governmental	3 years
12	29	Governmental	5 years
13	33	Governmental	27 years
14	29	Governmental	8 years
15	23	International NGO	13 years
16	94	International NGO	20 years
17	55	International NGO	5 years
18	26	International NGO	15 years
19	32	Local NGO	12 years

Table 1 describes each participant’s interview length, their affiliation, and years of experience.

Participants identified and embraced many opportunities to explore the country’s HIS for adolescent health. These opportunities represented crucial pathways towards achieving a more robust and effective system. This section delved into the eight key opportunities they described. These encompassed capacity building, streamlining, and digitizing the HIS, fostering collaboration across sectors, reshaping services based on health information, resourceful collaboration, empowering community engagement, particularly among adolescents, and implementing strategic actions for informed adolescent health. While these opportunities paved the way for transformative change, it’s also essential to acknowledge the HIS’s significant challenges, including resource constraints, data quality issues, and the imperative need for more comprehensive data sharing and utilization. The results explored the potential for progress and the challenges in strengthening the adolescent HIS.

### Opportunities

#### 1. Institutional and individual capacity building

Many participants expressed great confidence in the surveys the Palestinian Central Bureau of Statistics (PCBS) produced. They felt they were experienced in data collection and provided capacity building in the training and fieldwork supervision. They described that this helped in the institutional capacity building of a solid body trained in data collection and could provide quality data.

*“The international consultant came here locally to the country and conducted the training. Many of them are in the piloting stage, testing the tools themselves, modifying them, etc. So, again, this was joint work in which we were heavily engaged. So, we are confident in the results of this survey. We supported capacity building in the training and fieldwork supervision to ensure the quality of the data being gathered. To ensure that we have a harmonized understanding of the tool, the questions, and the data collection process, and we support the supervision during the data collection period, we jointly work with them on the data cleaning and analysis. We work side by side with PCBS.”–Participant ID #16*

As it is essential to provide institutional capacity building, the participants described that individual capacity building was also given. Many different organizations were sure to train their field workers and data collectors. They also monitored the data entry to ensure data quality. They described that, for the most part, the collected surveys were of good data quality.

*“We have field workers that are always in the field. They are always available… our field workers are trained beforehand. Then, we have project coordinators. They usually have one program manager and the program manager to the director to the unit director.” Participant ID #3*

#### 2. Digitalization of HIS

In the digitalization age, many participants described the government piloted digital records for both primary and secondary health facilities. This made it easier for patients and healthcare providers to provide better care and ensured that the needed information was available. The governmental participants described that the primary health care system used software called DHIS2 and Avicenna. Participants also mentioned that non-governmental organizations also used this software.

*“We have a national software and database for governmental and non-governmental hospitals, and they use the same system (Avicenna). This has made lives easier and made it easier for patients. They don’t have to carry their file to every doctor they see to ask them why they are here. Also, sometimes doctors write things that patients don’t understand, so it’s easier for the doctor to look at the patient’s history.” Participant ID #1*

#### 3. Connecting fragmentation

With the various types of healthcare providers and organizations working in adolescent health, it was tough to keep track of all the different activities, programs, and interventions offered in the country. Many discussed being part of the Palestinian Adolescent Health Coalition. They felt that the coalition was a great way to keep informed on the different adolescent health activities and help lessen the duplication of services. It also encouraged multi-sectoral collaboration as it brought together all the partners in adolescent health.

*“In 2018, we decided to see what institutions are working with or on adolescents. It was not efficient to see such hard work so scattered. So, we started and built the Palestinian Adolescent Health Coalition. Right now, there are more than 33 organizations, some of them are local NGOs that work with youth from Sharek to Juzoor to World Vision, as well as international organizations such as Save the Children, Y-peer, Tomorrow Youth in Nablus, UNICEF, WHO in Ramallah the institute-would like to join, the PMoH, the Ministry of Education all of us are in one hub. We meet regularly and hold an annual conference each year.” Participant ID #3*

#### 4. Reorienting services to overcome challenges

When asked about the availability of adolescent health data, a few participants described the lack of data due to the lack of adolescent health services. They described the opening and closing of a youth-friendly clinic in Hebron. In 2012, the PMoH formed a youth-friendly clinic in Hebron. After some time, the clinic closed. When asked about examples of what kind of clinics worked for adolescents, it was found that most family services operated in schools or universities provide adolescent health services. Many described the move of family services to their primary healthcare clinics by the PMoH. This way, the entire family could go to one place and receive the needed services. One of the participants was involved in creating the manual to address adolescent and youth health and hoped that, with this move, more adolescent health data could be collected.

*“A youth-friendly clinic was started, which did not work because everyone who went there obviously went to this. We thought this would not work; it should be integrated into the system. You go to the clinic and see someone you know; maybe he is there because his stomach hurts or his throat or he has something he wants to talk about. We made the job aid and manual that tackles everything related to youth health… We trained doctors, nurses, and midwives. We trained them in the manual and are now integrating it within primary healthcare so we can reach adolescents everywhere.” Participant ID #12*

Many described the hesitation of adolescents seeking health information from a reliable source or healthcare for fear of lack of privacy. Two participants described the creation of an app for adolescents to ask a doctor any question they wanted confidentially. They could write and ask about anything and receive a reliable response from the doctor in 24 hours. With this application, they could track topics that were asked, who used the app, and the gender/age of participants, but no identifying information of who asked the question. When asked about the health data retrieved from this app, they said it had yet to be analyzed.

#### 5. Smart collaboration to work with limited resources

Another opportunity for success was the identification of collaboration to work with limited resources. Being in a humanitarian country, funds were often limited and donor-based. One of the participants identified the need for adolescent health data. They found it was best to use the Global School Health Survey to collect adolescent health data but did not have the human resources or financial backing to collect the needed data. They were able to collaborate with other organizations in adolescent health, using their staff to administer the survey in schools, requiring minimal financing.

*“By working with others and using their staff to administer the survey in schools, it will not cost much to collect the data.” Participant ID #11*

#### 6. Empowerment of organizational program ownership

A few of the participants not working in the governmental sector addressed the need to include the governmental sector in any new program or intervention they hoped to launch. They felt that allowing the governmental sector to be a part of the planning and implementation of programs would help the programs and interventions succeed. This gave the government ownership of the programs for sustainability purposes.

*“It is important for the national partners to be the ones that implement our programs for sustainability.” Participant ID #6*

*“We work with our partners to include the government as much as possible to help implement programs.” Participant ID #5*

*a*. *Empowering adolescents*. Three of the participants described using adolescents and youth in what they stated was peer-to-peer learning. This was part of an international program called Y-peer. Knowing that Palestine’s context differed from other areas, they were sure to tread lightly in an effort not to offend cultural norms. Adolescents were trained by their peers in suggested topics such as gender roles, sexual health, and gender-based violence. After this training, they became part of the mobile clinics where they would use different games to spread awareness.

*“Members of the network joined after specialized training. They become a part of the mobile clinics funded by the United Nations Population Fund (UNFPA) in partnership with the Palestinian Medical Relief Society. They are a part of the mobile clinics… They can even do recreational activities for children there. In 2021, we did a workshop for adolescents. We transformed traditional games such as Monopoly and Snake and Ladders and used them to spread awareness about sexual and reproductive health.” Participant ID #17*

#### 7. Strategic actions for adolescents for information

*a*. *Forming a national strategy for adolescent health*. Many participants described the necessity of forming the Department of School and Adolescent and Youth Health by the PMoH. They also described that they took part in drafting the national strategy. The department’s vision, mission, and primary objectives were written for the next five years.

*“The original idea was established in 2019. After three years of working on the strategy with members of the Ministry of Health and outside partners, we recently launched the strategy (July 2022). After the launching, the Department of School and Adolescent and Youth Health was formed.” Participant ID #3*

*“We contributed to developing the Adolescent Health Strategy jointly with UNFPA*, *the Minister of Health*, *and other organizations*. *And now*, *we look forward to our new program cycle to sit together with the Ministry and other partners*. *The next priority is that we should all know what to contribute to*.*” Participant ID #12*

*b*. *Addressing adolescent needs*. A few of the participants working in NGOs described the establishment of the Department of School and Adolescent Health as what paved the way for other non-governmental and civil society organizations to review their mandates and include the adolescent age group as one of their priorities in some of their programs, especially regarding sexual and reproductive health. They found that adolescents sought certain information and formed certain behaviors and beliefs before they became adults.

*“Originally, our projects were geared towards youth. Recently, it has been changed to adolescents and youth.” Participant ID #17*

*“Our organization’s mandate looks at youth (18–35). One of the projects we are working on is sexual reproductive health (SRH). We did some baseline surveys and research, and we found that we needed to incorporate the ages of (14–18). This age group is going through puberty in schools and has access to reliable SRH information. On this basis, we included them in our SRH project.” Participant ID #18*

*“We started working with ages under 18 because we found that most negative behaviors and beliefs are formed before the age of 18*, *and we have a chance of changing these behaviors and beliefs while they are younger*.*” Participant ID #6*

### Challenges

Although the HIS in the West Bank has seen many improvements regarding adolescent health, it has also faced many challenges. Challenges included the high workload of staff, lack of health information specialists, limited resources, not a unified system in data collection, and lack of data on essential indicators, data quality, data sharing, and data sources and use.

#### 1. Staff shortage

Challenges were found in the high workload of staff, lack of health information specialists, lack of data on specific indicators, and incomplete data. The HIS was not given the attention that was needed. Many participants addressed the lack of staff for specific informational needs. For example, when something is not digital, an employee must enter the data later. One organization saw the need for an employee to run the HIS, but it was difficult to justify this need with a donor because it was donor-based. In the Ministry of Health, the health information department, which was in charge of all of the Ministry’s data in the West Bank, had only twelve employees.

“*I wish there was a person for data entry. We don’t have a person responsible, and we have so much information. This is a missing loop; with all this information and the research we made, we should have a huge database, but we can’t bring someone. We are project-based. Because I am working on that project and still need help with other projects. It is very hard to convince a donor that I need someone to create the database and do data entry, but I think this is needed because all the work we do gets missed.” Participant ID #3*

#### 2. Lack of health information specialists

One of the participants who had experience working in HISs revealed that one of the biggest problems faced by human resources was the lack of specialization in HISs in the country. Due to the lack of specialization, employees were working in areas where they were not entirely familiar with what the job exactly entailed. It was clarified there was this misunderstanding: when you refer to information systems, it was automatically equivalated to information technology (IT), when these are two completely different things. Whenever training was offered in HISs, management sent someone who worked in the IT department.

*“We do not focus on the specialization in information systems. When you go to the health statistics department in the Ministry of Health and find that the people who work there are not specialized in information systems but specialized in IT, there is a problem. It is also a problem when the person in charge doesn’t know that information systems are not equivalent to IT.” Participant ID #1*

#### 3. Resources

*a*. *Poor internet connectivity*. The internet was a significant resource in moving from paper records to electronic records. Some participants referred to the lack of internet in some rural areas as making it harder to digitalize the HIS. After collecting information, the employee was required to input the data into the electronic system later. The lack of internet has been addressed but has yet to be resolved.

*“There are 50–60 primary health clinics that are not digitalized because of connectivity to the internet. These are in Area C and do not have Jawwal or Ooreedo connections (cell towers). At my last meeting with the Ministry of Communication, they tried to fix the problem because there are schools with the same issues, and we are still waiting.” Participant ID #1*

#### 4. Not a unified system for data collection

Although the governmental sector was able to digitalize both the primary and secondary health facilities, they could not use the same data collection system; making it harder for them to be connected in a unified system. The primary health care, limited to reproductive health, used DHIS2, while the hospital used Avicenna medical software. They were currently working on how they would be able to unify them. Also, due to poor internet connectivity, paper-based reports and files were still being used.

*“Having one harmonized unified system within the Ministry itself has been a challenge, and so there are a lot of interventions from WHO, for instance, from our side, and from other partners regarding improving the HIS.” Participant ID #16*

#### 5. Indicators

*a*. *Input and outcome compared to impact indicators*. Several participants clarified that input indicators usually could be found when indicators were collected. They described having information for indicators such as the number of adolescents who received services, the prevalence of anemia, and obesity. They defined impact indicators as the indicators that give you the information picture of the situation. They acknowledged the importance of having impact indicators, which they knew they needed to collect.

*“We are trying to move towards impact-based indicators more than output or outcomes indicators because this widens our overall picture. Many times, we face difficulties in catching or not easy to track the impact (of our programs). This is not something that is easy; it has caused us to put different indicators into one.” Participant ID #2*

*b*. *Unified indicators for adolescent health*. Due to adolescent health being a newer area of concern, many of the participants were unsure of the indicators that they should be collecting. There was a consensus on the recommendation of a unified core indicator list. This list should be agreed upon among the organizations that work in adolescent health. It would help the organizations build their future programs or interventions and provide a way to measure and monitor success as well.

*“We need to prioritize which indicators we focus on, which ones are more relevant, and to publicize it to all organizations. This will help us design new projects, interventions, programs, and so on. To refer to these indicators and see that they are the most relevant to the Palestinian context. These (indicators) are more relevant to SDGs, and to the future interventions we want to make, we can unify them to all the organizations. Participant ID #2*

*c*. *Lack of data for essential indicators*. Most of the participants agreed that limited data was available regarding adolescent health. They agreed that this included the lack of data for essential indicators such as child labor indicators, disability, and mental health such as depression and anxiety. Limited data caused a lack of focus on these areas, resulting in insufficient programs for battling adolescents who dropped out of school and joined the labor force.

*“Learning of employment indicators would also be important to be measured and collected. It will influence our schools’ policies, maybe for the higher education system in Palestine. It would, as I said, influence me without labor laws in Palestine, etcetera, and I cannot deny that adolescents are dropping out of school and joining the labor force. Unfortunately, without necessarily the correct and healthy safe environment for them to work and that’s why we for instance, we are not focusing maybe enough on child labor in Palestine as we lack information on what is the size of adolescents in the labor force, for instance, in Palestine.” Participant ID #16*

#### 6. Data quality

Although the population-based surveys administered showed good data quality, there was a consensus that the data quality of hospital and primary health care records overall was not the best. One of the participants described it as hit or miss, unsure of the data quality. The data quality was thought to be because of the lack of monitoring of the data collection, resulting in incomplete or inaccurate data.

More than one participant mentioned one of the main challenges of using data from routine data was the consistency of incomplete data. One of the participants described the inability to use the collected information for research.

*“Once, I assessed the emergency rooms with the World Bank. I needed to take information from Avicenna; it’s horrible; you can’t get anything out of it, and they (health providers) barely enter anything, even when they enter, whatever they enter is for the sake of filling in text.” Participant ID #14*

Another participant described in reviewing files gave an example of some errors viewed in the available data.

“*When reviewing a cause of death, I noticed they had put a cause of death that was associated with females when his name was X, and he was 72 years old. We had to open the patient’s record to make sure of the cause of death.” Participant ID #11*

Technical, human, and system errors like this were common, and it took more resources than available to ensure the data was correct and complete.

#### 7. Data sharing

When asked about data sharing, many described the lack of a data-sharing system. All participants felt data sharing was essential but understood it was almost nonexistent. They also agreed that the PMoH should take the lead in formalizing a data-sharing system requiring any organization that provides health services to share its data with the PMoH.

*“We don’t mind sharing the data, but we don’t have the database, we don’t mind sharing anything, but we don’t have the database or the person that can be not based on a project.” Participant ID #3*

Three participants mentioned data sharing was problematic because organizations did not truly understand data ownership. They went on to discuss that it did not mean that you should collect the data and put it away under lock and key and not share it with anyone; it means you are the owners, and you must make sure the data is as accurate as possible, and it is used to be representative of the situation and proper credit for data collection is assigned.

*“We don’t have a problem with data sharing, but we have a problem with publishing our data without our knowledge or permission. We have to know what is published because, unfortunately, from our experience, we have seen the fabrication of data, so we must be aware of the data and results that come from us.” Participant ID #14*

*“I am not sure why more data is not shared; maybe every organization in their thinking and point of view (the data) should come out in their name*.*” Participant ID #9*

#### 8. Data sources and use

When asked how data was used to decide programs, many participants described the unavailability of adolescent health data. Many of the programs currently being run were organizational initiatives or donor-based programs. To justify donor-based programs, non-governmental agencies often perform a needs assessment or a baseline survey to determine if a need was seen. These assessments were not published and were for organizational purposes only.

## Discussion

In this study conducted in the West Bank, we identified opportunities and challenges within the Palestinian HIS concerning adolescent health. The opportunities encompassed capacity building, digitalization of health records, multi-sectoral collaboration, service reorientation based on health information, strategic actions for adolescents’ well-being, and enhancing community engagement. However, the study also unveiled critical challenges, including staff shortages, a lack of health information specialists, poor internet connectivity, non-unified data collection systems, insufficient data for essential indicators, incomplete or incorrect data, limited data sharing, and issues surrounding data ownership. Addressing these challenges is crucial for the use of the HIS for informed decision-making, evidence-based policies, and improved adolescent health outcomes in the West Bank.

As the priority for adolescent healthcare has grown in recent years within the country, it has become key to assess the capacity of the current HIS to manage the collection and dissemination of adolescent health data effectively. Our findings indicated that the HIS holds substantial potential, exemplified by its ability to employ data for service reorientation to address challenges faced by certain programs. This is a promising illustration of the impactful role that data utilization can play in shaping adolescent health initiatives. Furthermore, establishing family services within governmental primary healthcare clinics, where entire families can access comprehensive services in a single location, contributes significantly to safeguarding privacy and mitigating the stigma that often deters adolescents and youth from seeking care. This approach, proven successful among Latino populations in the United States, offers adaptable possibilities for other settings [29].

It was also found that implementing digital records for primary and secondary health facilities can help healthcare providers offer better care, ensuring that information is available when needed. Technology allows for the capture of data more quickly, lessens the chances of errors compared to manual entry, and helps organize information efficiently. This is a tool for a broader approach to change, and healthcare professionals must be open to this change [30]. Furthermore, no unified system in data collection within primary and secondary health facilities does not help link both systems. It was found that the primary and secondary health care centers do not share the same digital problems, so the exchange of information is not feasible. The DHIS2 system was developed to collect data from different levels of health facilities and users [31]. The software has been used in 46 countries and is known for its ease of use and adding new modules if needed [32]. To overcome these challenges and advance toward a more efficient HIS, healthcare professionals must embrace digital records integration while also addressing staff shortages and providing adequate health informatics training.

Staff shortages have resulted in the overload of current staff, with the need for data entry personnel. Additionally, there is a need to invest in health information programs and understand that this differs from IT specialists. The HIS involves more than just electronic records. With an increase in the use of computers and electronic records, it is essential that the staff receive the proper training in health informatics. Providing institutional and individual capacity building is essential for building a solid body of training in data collection and ensuring the data’s quality. Technology and e-health are vital to improving health and moving forward toward universal health coverage. A study by the WHO found that 60% of countries in the Eastern Mediterranean and African Region reported having access to training towards e-health [33]. A study done in Palestinian and Jordanian hospitals showed that 86% of the staff included in the study desired health informatics training [34].

It was also found there is a limited understanding of the importance of data and information among health professionals [30]. Healthcare professionals should also be trained in data entry and given incentives to provide good data quality entry [30]. Data quality was reflected in the lack of incentives and training and was also seen in other countries. In a review of the data quality of HISs in low and middle-income countries, no country showed a percentage of data quality higher than 50% [35]. The movement from paper-based reporting to electronic records plays a role in improving data quality [36]. Increasing the availability of training also helped increase data quality [37].

The unavailability of internet connectivity was a major challenge, especially in rural areas, making it harder to digitalize the HIS. A literature review showed that digital access nowadays is just as important as water and electricity connections. Communities are being punished for living in rural areas [38]. It was suggested that offline data entry and then connecting to the internet to load on the system would help overcome the lack of internet [39]. This was seen in the West Bank as well. Furthermore, no unified system in data collection within primary and secondary health facilities does not help with the linkage of both systems [40].

Data sharing was also viewed as a challenge to ensure that the data was not manipulated for preferred results and that the privacy of patients was maintained. This is seen as a challenge in most low and middle-income countries to ensure that data is anonymized and individual information is not illegally shared [41]. To help with the sharing of data and ensure ownership was not lost, an INDEPTH data depository was created. It was created to help low and middle-income countries share their data in a more structured way [42]. Another barrier found in data sharing amongst health professionals in Malawi, India, and Senegal was staff shortages and lack of incentives to fill in reports properly [43]. It was also found that the lack of knowledge-sharing culture also played a role in health professionals sharing health information.

Limited data is also available for essential adolescent health indicators. The lack of unified indicators for adolescent health is another challenge, with many participants unsure what indicators they should collect. Along with limited data, there was a sense of different definitions of the available indicators. It is essential to establish metadata standards in order to enhance documentation and facilitate the seamless integration of data across various countries, time periods, and sources [44]. Implementing even small changes to the HIS in the West Bank, despite the challenging environment, will significantly enhance the collection and utilization of adolescent health data and information.

### Strengths and limitations

This is the first study to look at adolescent health from an HIS perspective in the West Bank. Analyzing the HIS regarding adolescent health is the first step in improving the overall adolescent health system. Participants were eager to share and learn ways to improve their overall HIS. The researchers were based locally, and the native language was used for interviews. As a limitation of this study, member checking was not feasible despite employing peer debriefing to enhance data validation. The potential burden on participants, who were already involved in other project activities where the research results were being applied, deterred additional engagement. Conducting member checking would have imposed an undue strain on their time and availability, thus limiting this aspect of our validation process. Also, one of the limitations that were faced was the inclusion of only participants from the West Bank. Results cannot be generalized for the occupied Palestinian territory, as the Gaza Strip and East Jerusalem were not included in the analysis.

## Conclusion

In conclusion, this study has illuminated both the promising opportunities and persistent challenges within the Palestinian healthcare system’s HIS concerning adolescent health. While the system has demonstrated significant potential in capacity building, digitalization of health records, multi-sectoral collaboration, service reorientation, and enhancing community engagement, it is equally burdened by the daunting challenges of staff shortages, non-standardized data collection systems, insufficient data for essential indicators, and a lack of impact-based indicators. Furthermore, the limited culture of knowledge sharing remains a hurdle to realizing the full potential of HIS. To address these issues, we recommend urgent attention to staff shortages through comprehensive training programs for health information specialists, a concerted effort to standardize data collection systems, the development of a unified core indicator list for adolescent health, and the promotion of a robust knowledge-sharing culture among healthcare professionals and involved organizations. Embracing these recommendations will fortify the HIS and propel the Palestinian healthcare system towards improved adolescent health outcomes and more informed decision-making, despite resource limitations and complex humanitarian settings.

## Supporting information

S1 File Interview guide for key informant interviews. This interview guide was used to guide interviews with key informants.
(DOCX)

S2 File COREQ, the consolidated criteria for reporting qualitative studies. This checklist is a guide to reporting qualitative research.
(DOCX)

## Abbreviations

DHIS2: District Health Information Software 2
HIS: Health Information System
IT: Information Technology
NGOs: Non-Governmental Organizations
PCBS: Palestinian Central Bureau of Statistics
PMoH: Palestinian Ministry of Health
SRH: Sexual Reproductive Health
UNFPA: United Nations Population Fund
WHO: World Health Organization
