# Supplementary material for: Opportunities and challenges of using a health information system in adolescent health management: A qualitative study of healthcare providers’ perspectives in the West Bank, occupied Palestinian territory
Source: PLoS One. 2024 Aug 22;19(8):e0307207. doi: 10.1371/journal.pone.0307207 (PMC11340989; doi:10.1371/journal.pone.0307207)
Supplement: S1 File — This interview guide was used to guide interviews with key informants. (DOCX) [file pone.0307207.s001.docx]

**Supplement 1: Interview guide for key informant interviews**

**Exploring Stakeholder Perceptions on Informational Needs and Barriers of the Adolescent Health System in Palestine**

Background and Objectives

These key informant interviews are a part of a more significant project, “Strengthening of the Adolescent Health Information System in Palestine.” We have mapped the stakeholders involved in providing adolescent health or educational services. We collected information about the type of services, location, gender of the target population, collaboration/partnership with other stakeholders, and whether the services are specific to humanitarian and fragile settings. Institutions were contacted to verify the information posted on their website.

We now hope to identify the following objectives:

1. Identify critical stakeholders involved in the adolescent health system and assess their roles in the health information system
   1. Identify the main adolescent health programs
2. Understand the flow of information across the system
   1. Evaluate the health information exchange (How is the information being shared?) (If sharing does not happen, why?) Barriers to sharing information?
3. Assess the different stakeholder’s informational needs
   1. Identify the perceived usability of the information (How do they use the data from the system?) (Why don’t they use the information?)
   2. Identify stakeholder information-seeking styles. (Where do they get their information from?)

Methodology:

The key informant interviews will include senior management, funders, data managers, and select personnel from regional offices involved in data collection. We will identify the key informants using a purposive sampling method and interview only people involved in the health information system process. These interviews will complement the results obtained from the desk review and system assessment. The interviews will include questions about the existing system, the follow of data collection, assessment, storage, analysis, reporting, and policy formulation. We will ask questions related to challenges facing the system and possible solutions from the key informant’s point of view. The interview guide has been attached for ethical approval. All interviews will be transcribed and stored for seven years, with only people; working on the project will have access to these transcriptions.

The following is an interview guide for each of the different types of stakeholders to be interviewed. Each stakeholder will be asked for informed consent before starting the interview.

**Informed Consent**

**Exploring Stakeholder Perceptions on Informational Needs and Barriers of the Adolescent Health System in Palestine**

This study is part of a project to understand better the health information system used in Palestine, with a particular focus on adolescent health. We want to discuss the different components one might encounter when collecting any data. Your participation is voluntary, and please do not hesitate to stop or skip a question or end the interview at any time. The information collected will be treated with confidentiality, and your name will be coded to protect your name and will not appear in the final report. There are no known risks to participating in this interview, and it should take approximately an hour in length. People who participate in this study may better understand the perceived needs of the adolescent health information system in Palestine. We would like to record the interview if that is okay with you. You can ask to turn the recording off at any time if needed. The recorded interview will be erased after completing the research, and only transcriptions will be stored for seven years. If you give us your oral consent, we would like to start.

هذه دراسة جزء من مشروع أكبر لفهم أفضل لنظام المعلومات الصحية المستخدم في فلسطين، مع التركيز بشكل خاص على صحة المراهقين. نود أن نناقش معك المكونات المختلفة عند جمع أي نوع من البيانات. مشاركتك طوعية ويرجى عدم التردد في التوقف أو تخطي أي سؤال أو إنهاء المقابلة في أي وقت. سيتم التعامل مع المعلومات التي تم جمعها بسرية ولن يظهر اسمك في التقرير النهائي. نود تسجيل المقابلة، إذا كان هذا مناسبًا لك. يمكنك طلب إيقاف التسجيل في أي وقت إذا لزم الأمر. سيتم محو المقابلة المسجلة بعد الانتهاء من البحث. إذا أعطيتنا موافقتك الشفوية، نود أن نبدأ.

**Funders**

Please tell me a little about yourself. How long have you worked at ________?

1. What is the process taken to decide on what is funded?
   1. ما هي العملية المتبعة لاتخاذ قرار بشأن ما يتم تمويله؟
2. What role does data play in funding projects?
   1. ما هو الدور الذي تلعبه البيانات في عملية تمويل المشاريع؟
3. What kind of data is expected from funded projects?
   1. ما نوع البيانات المتوقعة من المشاريع التي يتم تمويلها؟
4. What kind of feedback is given on the collected data?
   1. ما نوع التغذية الراجعة على البيانات التي يتم جمعها؟
5. What kind of policies are in place to guide the communication and sharing of data with external users?
   1. ما نوع السياسات المعمول بها لتوجيه الاتصال ومشاركة البيانات مع المستخدمين الخارجيين؟
6. Do you think data sharing is essential?
   1. هل تعتقد أن مشاركة البيانات مهمة؟
7. What are some of the barriers or strengths to data sharing? (based on how the above is answered)
   1. ما هي المعيقات أو نقاط القوة في مشاركة البيانات؟ (بناءً على كيفية الرد على ما ورد أعلاه)
8. What would you suggest would help with overcoming these barriers?
   1. ما الذي تقترحه من شأنه أن يساعد في التغلب على هذه المعيقات؟

**Data Collectors (Producers)**

Please tell me a little about yourself. How long have you worked at ________?

1. What is the nature of your work?
2. Are you given telephone, internet, and email access? (
   1. هل يتم منحك الهاتف والإنترنت والبريد الإلكتروني؟
3. Do you use computers or paper to report data?
   1. هل تستخدم أجهزة الكمبيوتر أو الورق لاعداد تقارير البيانات؟
   2. If paper, what is the process followed after data is collected?
      1. في حالة الورق، ما هي العملية المتبعة بعد جمع البيانات؟
4. Do you think the software is user-friendly? (if computers are used)
   1. هل تعتقد أن البرنامج سهل الاستخدام؟
   2. Why or why not?
      1. لماذا أو لماذا لا؟
5. Is there any equipment you wish was available, and do you think it would make it easier to collect data?
   1. هل هناك أي معدات تتمنى لو كانت متوفرة لديك، وتعتقد أنها ستجعل جمع البيانات أسهل؟
6. What kind of training have you received in the past six months?
   1. ما هو نوع التدريب الذي تلقيته في الأشهر الستة الماضية؟
7. Tell me about the process the information goes through… What kind of forms do you have to fill out? (names of the forms)
   1. أخبرني عن العملية التي تمر بها المعلومات …… ما نوع النماذج التي يجب عليك تعبئتها؟ (أسماء النماذج)
   2. Any related to adolescents?
      1. أي منها متعلق بالمراهقين؟
8. What kind of assistance is available regarding the databases and software?
   1. ما نوع المساعدة المتوفرة لك فيما يتعلق بقواعد البيانات والبرامج؟
9. What role do you play in designing the data collection forms?
   1. ما هو الدور الذي تلعبه في تصميم نماذج جمع البيانات؟
   2. Indicators?
      1. المؤشرات؟
10. Who is responsible for filling in the HMIS monthly reports?
    1. من المسؤول عن ملء التقارير الشهرية لنظام إدارة المعلومات الصحية (HMIS)؟
11. Why do you think this data is collected?
    1. لماذا تعتقد أنه يتم جمع هذه البيانات؟
12. What kind of motivation is given to complete the data collection?
    1. ما هو نوع الحافز الذي يتم تقديمه لإستكمال جمع المعلومات؟
13. What kind of monitoring is done to ensure data quality?
    1. ما نوع المراقبة التي يتم إجراؤها لضمان جودة البيانات؟
14. What are some of the challenges faced in ensuring good quality data?
    1. ما هي التحديات التي تواجه ضمان جودة عالية للبيانات؟
15. What do you think are opportunities for improving data quality?
    1. ما هي الفرص المتاحة لتحسين جودة البيانات برأيك؟
16. What kind of feedback is given on the data that you collect?
    1. ما نوع التغذية الراجعة المقدمة لك على البيانات التي تجمعها؟
17. Are you confident that the data collected is essential?
    1. هل أنت واثق من أن البيانات التي تم جمعها مهمة؟
18. What are the main challenges regarding collecting and the quality of data you receive?
    1. ما هي التحديات الرئيسية المتعلقة بجمع وجودة البيانات التي تتلقاها؟
    2. What solutions do you suggest?
       1. ما الحلول التي تقترحها؟
19. Do you have anything else you would like to share?

**National Representatives, Policymakers (Users)**

Please tell me a little about yourself. How long have you worked at ________?

1. What kind of training did you provide to your employees in the past six months?
   1. ما نوع التدريب الذي قدمته لموظفيك في الأشهر الستة الماضية؟
2. What kind of motivation is given to complete the data collection?
   1. ما هو الحافز لإستكمال جمع المعلومات؟

**Data Demand and Use**

1. What kind of program decisions do you regularly have to make?
   1. ما نوع القرارات التي تتعلق بالبرنامج التي يتعين عليك اتخاذها بانتظام؟
2. What data is available for these decisions?
   1. ما هي البيانات المتوفرة لهذه القرارات؟
3. What kind of information do you find necessary to inform a decision?
   1. ما نوع المعلومات التي تجدها ضرورية لاتخاذ قرار؟
4. How do you use the data for these decisions?
   1. كيف تستخدم البيانات لهذه القرارات؟
5. How do you retrieve the data for use?
   1. كيف تسترجع أو تستخرج البيانات لاستخدامها؟
6. Where would you think of using data?
   1. ما رأيك في استخدام البيانات؟
7. What do you think can be done to improve the use of data?
   1. ما الذي تعتقد أنه يمكن القيام به لتحسين استخدام البيانات؟
8. What are the main challenges regarding the design of the HMIS?
   1. ما هي التحديات الرئيسية فيما يتعلق بتصميم نظام المعلومات الصحية HMIS؟
9. How often do meetings occur regarding the use of the information?
   1. كم مرة تعقد الاجتماعات فيما يتعلق باستخدام المعلومات؟
10. Do you have any reports or meeting notes that discuss how the information will be used?
    1. هل لديك أي تقارير أو محاضر اجتماعات تناقش كيفية استخدام المعلومات؟
    2. What kind of follow-up action has taken place regarding these meetings?
       1. ما نوع إجراءات المتابعة التي تم اتخاذها فيما يتعلق بهذه الاجتماعات؟
11. Giving examples, how does the Ministry use the information in the health management system?
    1. إعطاء أمثلة كيف تستخدم الوزارة المعلومات في نظام الإدارة الصحية؟
12. What are the main challenges in using data collected in adolescent health?
    1. ما هي التحديات الرئيسية في استخدام البيانات التي تم جمعها في مجال صحة المراهقين؟
13. Any suggestions on this can be resolved.
    1. يمكن حل أي اقتراحات بشأن هذا؟
14. Is there information that you wished was available for use? (Academics and researchers)
    1. هل هناك معلومات كنت تتمنى ان تكون متاحة للاستخدام؟ (أكاديميون وباحثون)
15. How do you ensure that all patient information remains private?
    1. كيف تتأكد من سرية معلومات المريض؟

**Data Sharing**

1. Can you give me an example of how data is shared within the Ministry?
   1. هل يمكن أن تعطيني مثالاً على كيفية مشاركة البيانات داخل الوزارة؟
   2. With NGOs?
   3. هل يمكن أن تعطيني مثالاً على كيفية مشاركة البيانات مع المنظمات الغير حكومية؟
   4. UNRWA
   5. هل يمكن أن تعطيني مثالاً على كيفية مشاركة البيانات مع وكالة الغوث وتشغيل اللاجئين؟
   6. Referral services
      1. خدمات التحويلات
2. What kind of policies are in place to guide the communication and sharing of data with external users?
   1. ما نوع السياسات المعمول بها لتوجيه الاتصال ومشاركة البيانات مع المستخدمين الخارجيين؟
3. Do you think data sharing is essential?
   1. هل تعتقد أن مشاركة البيانات مهمة؟
4. What are some of the barriers or strengths of data sharing? (based on how the above is answered)
   1. ما هي المعيقات أو نقاط القوة في مشاركة البيانات؟ (بناءً على كيفية الرد على ما ورد أعلاه)
5. What would you suggest would help with overcoming these barriers?
6. ما الذي تقترحه من شأنه أن يساعد في التغلب على هذه المعيقات؟

**Data Sources/Management**

1. What are the primary sources of information?
   1. ما هي المصادر الأولية للمعلومات؟
2. What is the time period between data collection and publication of statistics?
   1. ما هي الفترة الزمنية بين جمع البيانات ونشر الإحصائيات؟
3. How is the data stored?
   1. كيف يتم تخزين البيانات؟
4. What current data sources, or quickly collectible information, could be used to measure adolescent health indicators?

ما هي مصادر البيانات الحالية ، أو المعلومات التي يمكن جمعها بسرعة ، والتي يمكن استخدامها لقياس مؤشرات صحة المراهقين؟

- 1. National/regional data sets/surveys that provide adolescent health indicators?
     1. (مجموعات / استقصاءات) بيانات (وطنية / إقليمية) يمكن أن توفر مؤشرات صحة المراهقين؟
  2. Information that can be collected through a brief policy survey and review of policy documents?
     1. المعلومات التي يمكن جمعها من خلال مسح موجز للسياسة ومراجعة وثائق السياسة؟

1. What are the current processes and challenges for the Ministry of Health and other key stakeholders?

ما هي العمليات والتحديات الحالية التي تواجه وزارة الصحة وأصحاب المصلحة الرئيسيين الآخرين؟

1. data availability on the adolescent health indicators
   1. توافر البيانات حول مؤشرات صحة المراهقين
2. monitoring systems used to measure current adolescent health indicators
   1. نظم الرصد المستخدمة لقياس مؤشرات صحة المراهقين الحالية
3. Is data recorded with sufficient details to measure relevant indicators?
   1. هل البيانات مسجلة بتفاصيل كافية لقياس المؤشرات ذات الصلة؟
   2. How so?
      1. كيف ذلك؟
4. Are there clearly defined procedures to identify discrepancies in reports?
   1. هل توجد إجراءات محددة بوضوح لتحديد التناقضات في التقارير؟
   2. Give me an example
      1. , , , أعطني مثال

**Data Dissemination**

1. What percentage of facilities are represented in the HMIS information?
   1. ما هي النسبة المئوية للمرافق الممثلة في معلومات نظام معلومات الإدارة الصحية؟
2. Does the health ministry have the most recent data available on its website?
   1. هل لدى وزارة الصحة أحدث البيانات المتوفرة على موقعها الإلكتروني؟
3. What kind of information is published?
   1. ما نوع المعلومات المنشورة؟
4. Where do you get your information about adolescents from?
   1. من أين تحصل على معلوماتك عن المراهقين؟
